# Onset of Oral Lichenoid Lesions and Oral Lichen Planus Following COVID-19 Vaccination: A Retrospective Analysis of about 300,000 Vaccinated Patients

**DOI:** 10.3390/vaccines10030480

**Published:** 2022-03-20

**Authors:** Moritz Hertel, Andrea-Maria Schmidt-Westhausen, Stephanie Wendy, Max Heiland, Susanne Nahles, Robert Preissner, Saskia Preissner

**Affiliations:** 1 Department of Oral and Maxillofacial Surgery, Charité—Universitätsmedizin Berlin, Corporate Member of Freie Universität Berlin, Humboldt-Universität zu Berlin, and Berlin Institute of Health, Augustenburger Platz 1, 13353 Berlin, Germany; moritz.hertel2208@gmail.com (M.H.); stephanie.wendy@charite.de (S.W.); max.heiland@charite.de (M.H.); susanne.nahles@charite.de (S.N.); 2 Department of Periodontology, Oral Medicine and Oral Surgery, Charité—Universitätsmedizin Berlin, Corporate Member of Freie Universität Berlin, Humboldt-Universität zu Berlin, and Berlin Institute of Health, Aßmannshauser Str. 4–6, 14197 Berlin, Germany; schmidt-westhausen@charite.de; 3 Institute of Physiology and Science-IT, Charité—Universitätsmedizin Berlin, Corporate Member of Freie Universität Berlin, Humboldt-Universität zu Berlin, and Berlin Institute of Health, Philippstr. 12, 10115 Berlin, Germany; robert.preissner@charite.de

**Keywords:** oral lichenoid lesion, oral lichen planus, adverse drug reaction, COVID-19 vaccine, SARS-CoV-2, real-world data

## Abstract

Introduction: Onset of oral lichenoid lesions (OLL) or oral lichen planus (OLP) can be rare adverse reactions to vaccines. Recently, the first solitary cases were reported after COVID-19 vaccination. The aim of the present study was to assess if an increased frequency of OLL/OLP can be found after COVID-19 vaccination within a large real-world cohort. It was assumed that the incidence of OLL/OLP was significantly higher in subjects who received COVID-19 vaccine (cohort I) compared to individuals who were not vaccinated (cohort II). Patients and Methods: Initial cohorts of 274,481 vaccinated and 9,429,892 not vaccinated patients were retrieved from the TriNetX database (TriNetX, Cambridge, Massachusetts, USA), and matched for age, gender and the frequency of use of non-steroidal anti-inflammatory drugs, beta blockers, and angiotensin-converting enzyme inhibitors. Results: After matching each cohort, we accounted for 217,863 patients. Among cohort I, 146 individuals had developed OLL/OLP within 6 days after COVID-19 vaccination (88 and 58 subjects had received mRNA- and adenovirus vector-based vaccines), whereas in cohort II, 59 patients were newly diagnosed with OLL/OLP within 6 days after having visited the clinic for any other reason. The risk of developing OLL/OLP was calculated as 0.067% vs. 0.027%, for cohorts I and II, whereby the risk difference was highly significant (*p* < 0.001; log-rank test). RR and OR were 2.475 (95% CI = 1.829; 3.348) and 2.476 (95% CI = 1.830; 3.350), respectively. Discussion: The hypothesis was confirmed. Accordingly, the obtained results suggest that the onset of OLL/OLP is a rare adverse drug reaction to COVID-19 vaccines, especially to mRNA vaccines. Thus far, it remains unknown if specific components of the formulations cause a type IV hypersensitive reaction corresponding to OLL, or if the immune response post vaccination triggers a T cell-driven autoimmune reaction directed against the basal layer of keratinocytes of the oral mucosa in terms of OLP. Although OLL and OLP are both classified as premalignant lesions, spontaneous remission may be expected over time, at least in the case of OLL. Therefore, the presented findings should not place any limitation toward the use of COVID-19-vaccines in broad levels of the population.

## 1. Introduction

Since the first cases arose in 2019, the coronavirus disease (COVID-19) caused by the severe acute respiratory coronavirus 2 (SARS-CoV-2) became a worldwide pandemic, and it is widely seen as today’s major challenge to the world’s healthcare systems [1]. A potential relief has been provided by the development of the SARS-CoV-2 (COVID-19) vaccines. Most of the sera are based on the presentation of the viral spike protein to the hosts immune system leading to active immunization by inducing an antigen-specific humoral response, specifically the formation of neutralizing antiviral immunoglobulins. The active agents are either mRNA coated in lipid nanoparticles (LNP) (e.g., BNT162b2 (BioNTech/Pfizer) and mRNA-1273 (Moderna)) or adenovirus vectors (e.g., ChAdOx1 nCoV-19 (Astra Zeneca), Ad26.COV2.S (Johnson & Johnson) and Gam-COVID-19-Vac (Gamaleya National Centre of Epidemiology and Microbiology)). Different from this approach, inactivated SARS-CoV-2 is available for vaccination as well (CoronaVac (Sinovac)). Clinical trials have shown both high reactivity and protection against COVID-19, especially regarding severe courses of the disease. Furthermore, safety profiles were found to be acceptable, despite minor adverse effects such as pain, fever, chills and fatigue having been reported [2,3,4,5,6,7,8,9,10]. However, severe adverse drug reactions (ADR) and adverse drug events (ADE) have emerged as new obstacles to the efforts to bring the pandemic to an end. In this regard, eosinophilic lung disease, cerebral venous sinus thrombosis, (CVST) pulmonary embolism and vaccine-induced immune thrombocytopenia (VITT) were found to be associated with the use of COVID-19 vaccines, especially the adenovirus vector-based sera [11,12,13,14,15,16,17]. Due to the fact that all COVID-19 vaccines have been produced, tested and delivered at unparalleled speed, more undesirable long-term effects as well as rare adverse events might become evident over time. Although reports on risks and complications may contribute to vaccine hesitancy, it is of uncontroversial ethical relevance that potential ADRs are well investigated and reported to the public, especially as a vast majority might choose vaccination over no vaccination due to the overweight of advantages.

**Figure 1:**
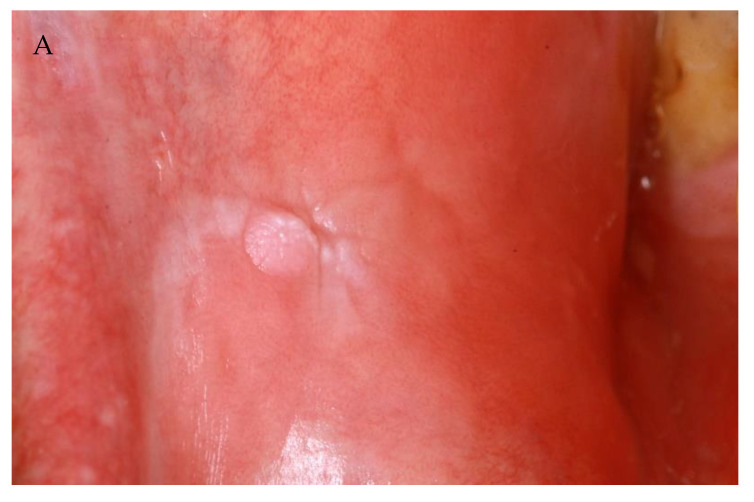

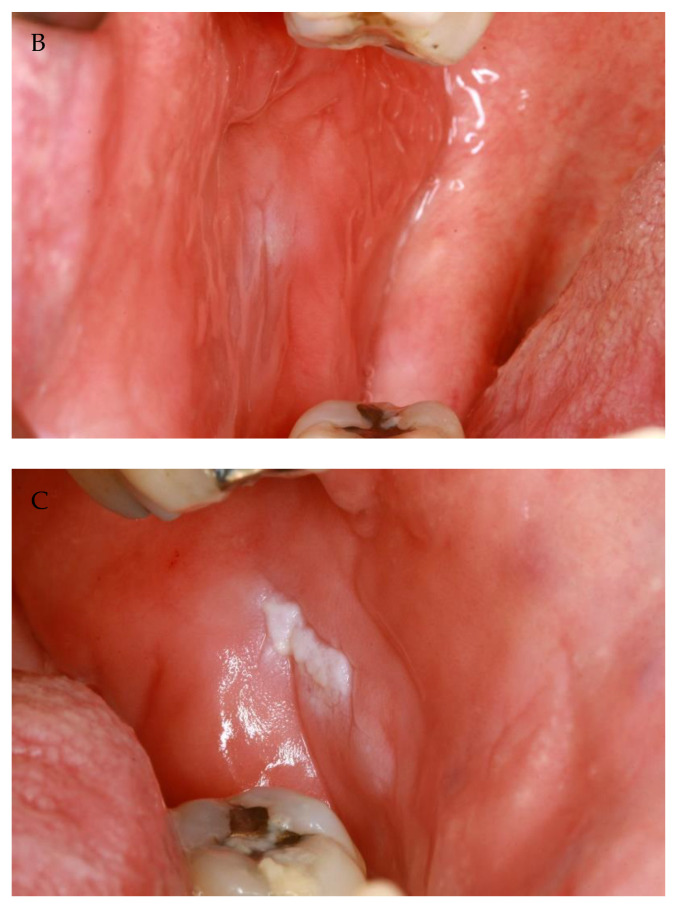
(**A**–**C**): Fifty-year old male with bilateral whitish papules and plaques of the buccal mucosa, which had appeared nine days after having received a second dose of mRNA-LNP spike protein BNT162b2.

The subject was brought into the focus of the authors when two patients presented themselves in the Department of Oral and Maxillofacial Surgery at the Charité—Universitätsmedizin Berlin (Head: Univ.-Prof. Dr. Dr. Max Heiland) after having developed multifocal oral lichenoid lesions (OLL) following COVID-19 vaccination. In September 2021, a 50-year-old male presented with bilateral whitish papules and plaques of the buccal mucosa (Figure 1A–C), which had appeared nine days after having received a second dose of mRNA-LNP spike protein BNT162b2. Eleven days later, a 57-year-old female was referred to the clinic for having developed whitish striae in the upper and lower vestibules 14 days after application of a second course of the same vaccine. Both subjects underwent biopsies, whereby the suspicion of oral lichenoid lesions (OLL)/oral lichen planus (OLP) was confirmed histologically.

Lichen planus (LP) is an autoimmune disease of unknown cause. It potentially affects the skin and/or the mucous membranes, including the oral mucosa. Different from LP/OLP, OLL is a type IV hypersensitive reaction toward noxious agents, such as corrosion products, or certain medication, e.g., non-steroidal anti-inflammatory drugs (NSAIDs), beta-blockers and angiotensin-converting enzyme (ACE) inhibitors. Therefore, OLL is termed lichenoid drug eruption as well [18]. Neither from clinical nor from histopathological characteristics can OLP and OLL be safely distinguished. As a consequence, we refer to both entities as OLL/OLP hereinafter. Efflorescences of both OLP and OLL vary from whitish lesions, specifically striae, plaques and papules to reddish alterations, which correspond to atrophy, erosion/ulceration or bullae. These lesions can occur solely or in any combination. The typical histopathology of OLP and OLL is characterized by an immunologic reaction dominated by CD8+ cytotoxic T cells, which directs against the basal layer of keratinocytes [19]. Hence, apoptotic cells can be found within the oral squamous epithelium (so-called Civatte bodies). Lichen planus and lichenoid drug eruption were previously described as extremely rare adverse reactions to vaccination, especially related to hepatitis B vaccine [20]. Drago and Rebora assumed that HBsAg and a sensitizing protein S play a role in the pathogenesis of OLL/OLP secondary to hepatitis B vaccination. The authors emphasized that protein S provides epitopes similar to keratinocytes, which might trigger an autoimmune response driven by cytotoxic T lymphocytes [21]. Furthermore, hepatitis B and rabies vaccination were observed to be associated with pediatric LP [22]. Regarding COVID-19 vaccination, an associated onset of cutaneous LP was recently reported by Hiltun et al., Merhy et al., Piccolo et al., as well as Belina et al. [23,24,25,26]. McMahon and co-workers found four cases with “lichen-like” histopathologic pattern among biopsies of 803 cutaneous reactions to COVID-19 vaccines [27]. A subsequently performed search of the recent literature revealed two reports of oral lichen planus following COVID-19 vaccine [28,29]. One male patient had received vector-based Ad26.COV2.S [28], whereas in the second report, “COVID-19 vaccine” was not further specified [29].

The aim of the present study was to investigate if an association between COVID-19 vaccination and the onset of OLL/OLP can be found not only in individual cases, but in a larger cohort based on real-world data. It was hypothesized that the incidence of OLL/OLP was significantly higher in patients who received COVID-19 vaccination compared to subjects who were not vaccinated.

To access data on the subject, the TriNetX Global Health Research Network (TriNetX, Cambridge, Massachusetts, USA) appeared to be appropriate, as it provides real-world data in high numbers. Medical records of more than 250 million patients have been implemented into the database by October 2021. TriNetX is a research network fed with clinical data by over 120 health care organizations (HCO) from 19 countries. Its intent is to connect healthcare institutes and contract research sites and biopharmaceutical companies to access longitudinal medical data, and to provide state-of-the-art analytics. It has already been used to research the COVID-19 pandemic [30,31].

## 2. Patients and Methods

### 2.1. Inclusion and Exclusion Criteria

TriNetX was accessed on October 24th, 2021, and the eligibility period was limited back to December 1st, 2020. The database was initially searched for both patients who had received at least one intramuscular injection of mRNA LNP- or adenovirus vector-based COVID-19 vaccine, and individuals who were not vaccinated against COVID-19. The initial cohorts together accounted for over 40 million subjects, which by far is above the capacity of the analysis tools of TriNetX. Hence, further inclusion criteria were defined as a visit of the HCO for evaluation and management services, and a most recent body mass index (BMI) value of 19–30 kg/m^2^. 

### 2.2. Matching Process

**Figure 2:**
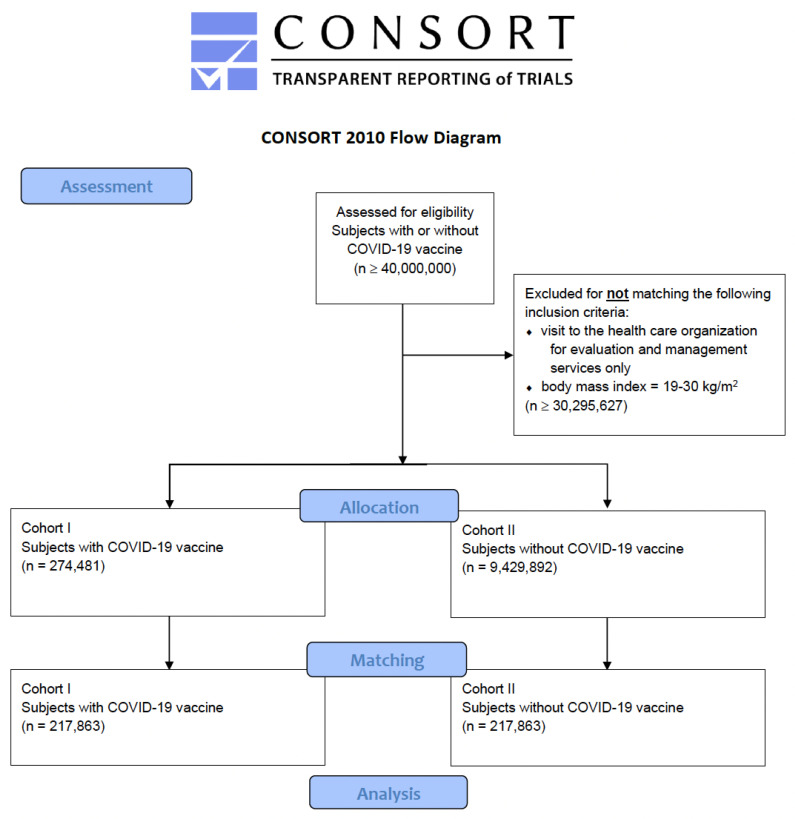
Modified CONSORT flow chart. Note that the number of eligible subjects was ≥40,000,000, which is above the analysis capacity of TriNetX. As a consequence, additional inclusion criteria were necessary to restrict the enclosure of patients.

Stratified and balanced sub-cohorts across current age and gender distribution, as well as the frequencies of the use of NSAIDs, beta-blockers and ACE inhibitors within the last three months before visiting the HCO, were retrieved from the initial cohorts as shown in Figure 2 in order to mitigate confounder bias via propensity score.

### 2.3. Statistical Analysis

After defining the primary outcome as “onset of OLL/OLP” within 6 days after COVID-19 vaccination regarding cohort I, and 6 days after visit of the HCO for any other reason for cohort II, Kaplan–Meier analysis was performed, and risk ratio (RR) as well as odds ratio (OR) were calculated for the respective groups. Statistical analysis was performed applying the log-rank test, whereby *p* ≤ 0.05 was defined as significance threshold.

## 3. Results

According to the inclusion and exclusion criteria, 274,481 and 9,429,892 individuals were eligible for cohorts I and II, respectively. After matching each cohort, we accounted for 217,863 patients. The demographic characteristics and the frequencies of the use of NSAIDs, beta-blockers and ACE inhibitors are displayed in Table 1. Despite the described matching process, a difference in the proportion of the subjects using NSAIDs remained (n cohort I: 48769 (22.39%) vs. cohort II: 47993 (22.03%); *p* = 0.046).

**Figure 3:**
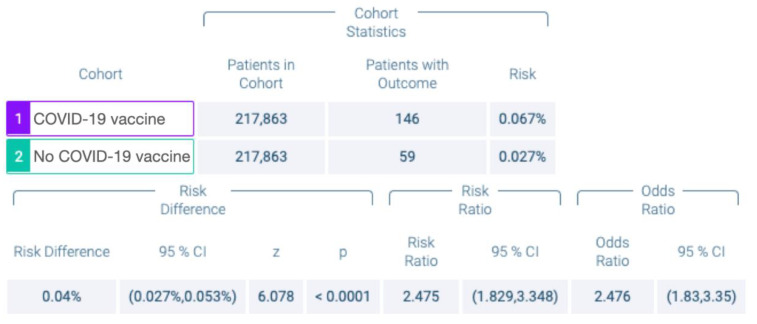
Number of patients with and without COVID-19 vaccination and risk of onset of oral lichen planus (OLP)/oral lichenoid lesions (OLL) within 6 days.

Among the subjects of cohort I, 146 individuals had developed OLL/OLP within 6 days after COVID-19 vaccination. Eighty-eight and 58 subjects had received mRNA LNP- and adenovirus vector-based vaccines. In cohort II, 59 patients were newly diagnosed with OLL/OLP within 6 days after having visited the HCO for any other reason. Accordingly, the risk of developing OLL/OLP was calculated as 0.067% vs. 0.027%, for cohorts I and II. The obtained risk difference of 0.04% was statistically highly significant (*p* < 0.001; 95% confidence interval (CI) = 0.00027; 0.00053). RR and OR were 2.475 (95% CI = 1.829; 3.348) and 2.476 (95% CI = 1.830; 3.350), respectively (Figure 3).

## 4. Discussion

The aim of the present study was to evaluate if the frequency of the onset of OLL/OLP was higher in patients who were immunized against COVID-19 (cohort I) than in individuals who were not vaccinated (cohort II). It was expected that the incidence of OLL/OLP was significantly higher among cohort I compared to cohort II. The hypothesis was confirmed referring to a 6-day period after vaccination/visit of the HCO. Accordingly, OLL/OLP appears to be a potential adverse drug reaction to COVID-19 vaccines, especially against mRNA LNP. However, the presented analysis found cases of newly diagnosed OLL/OLP in which adenovirus vectors had been administered as well. It may therefore be carefully assumed that the presentation of the viral spike protein to the hosts immune system might play a role in the pathological mechanism, causing OLL/OLP following COVID-19 vaccination. Despite this evidence, it remains unknown which exact component of the vaccines might be responsible for causing OLL/OLP. Furthermore, future studies are required to reveal the underlying pathological mechanisms. In the case of OLL, a certain cause is needed to unleash a type IV hypersensitive reaction, which might directly be an ingredient of the formulation. As for OLP, the immune stimulation following vaccination might presumably trigger a T cell-driven autoimmunologic reaction against the basal layer of keratinocytes of the oral mucosa. In accordance, long-term immunologic response to Ad26.COV2.S, including CD4+ and CD8+ T cell activation was shown inter alia by Alter et al. [32]. It has furthermore been discussed that the pathogenesis of COVID-19 might theoretically be enhanced by the presence of subneutralizing or cross-reactive nonneutralizing antibodies through ADRs/ADEs [33,34].

Despite the fact that OLL and OLP can cause complaints through erosion, ulceration or formation of bullae both are classified as premalignant lesions with an augmented risk of transformation into an oral squamous cell carcinoma (OSCC) [18]. Therefore, the presented findings implicate a potential risk of severe secondary consequential morbidity. However, spontaneous remission may be expected, at least in the case of OLL, which is why the risk of formation of OSCC can be cautiously estimated as being extremely low.

Along with the retrospective nature of the study, come certain limitations, which further studies may address, as outlined in the introduction, OLL and OLP cannot be distinguished, neither from the clinical presentation nor from histopathology. Thus, it remains uncertain if the recorded cases were OLL or OLP. If clinical follow-up would reveal a spontaneous remission over time, OLL could be diagnosed with relative certainty. In contrast, OLP/LP can be distinguished from OLL through the presence of cutaneous manifestations. Prospective clinical studies may consider differentiating between both entities. Thus far, it can be assumed that the majority of the lesions were OLL, as it is known to be associated with the use of different types of medication [18]. In this context, it needs to be discussed that the applied matching process could not completely eliminate the distribution difference in the frequency of the use of NSAIDs between both cohorts. As the percentage of patients using NSAIDs was higher in the cohort I (22.39 vs. 22.03%; *p* = 0.046) this might have at least contributed to the obtained results. To overcome the expressed limitations, future research might consider using a prospective approach to evaluate if the presented results can be confirmed thus far. 

## 5. Conclusions

The results obtained from real-world data suggest that the onset of OLL/OLP is a rare adverse drug reaction to COVID-19 vaccines, especially to mRNA LNP. However, spontaneous remission may be expected over time, at least in the case of OLL. Hence, the findings of the present study should not place any limitation toward the use of COVID-19 vaccines in broad levels of the population.

## Figures and Tables

**Table 1:** Demographic characteristics and the frequencies of the use of NSAIDs, beta-blockers and ACE inhibitors of the cohorts I and II after matching process.

	Before Matching				After Matching			
	Cohort I	Cohort II	*p*	Standardized Mean Difference	Cohort I	Cohort II	*p*	Standardized Mean Difference
**Number of patients (n)**	274,481	9,429,892			217,863	217,863		
**Female**	155,976 (56.85%)	5,017,913 (53.24%)	<0.001	0.0726	122,267 (56.12%)	121,547 (55.80%)	0.028	0.0066
**Male**	118,447 (43.15%)	4,409,276 (46.76)	<0.001	0.0725	95,555 (43.88%)	96,080 (44.20%)	0.109	0.0048
**Mean current age**	54.14	45.49	<0.001	0.3902	53.10	53.00	0.145	0.0044
**Standard deviation**	21.43	22.87			21.81	22.54		
**Minimum**	12	0			12	12		
**Maximum**	90	90			90	90		
**Use of:**								
**NASIDs**	78,580 (28.62%)	269,470 (2.86%)	<0.001	0.7565	48,769 (22.39%)	47,993 (22.03%)	0.046	0.0085
**beta-blockers**	82,560 (30.08%)	307,846 (3.27%)	<0.001	0.7710	38,832 (17.82%)	38,371 (17.61%)	0.067	0.0055
**ACE inhibitors**	50,575 (18.43%)	181,469 (1.92%)	<0.001	0.5673	28,638 (13.15%)	28,700 (13.17%)	0.781	0.0008

NSAIDs, non-steroidal anti-inflammatory drugs; ACE, angiotensin-converting enzyme.

## Data Availability

Raw data is available upon reasonable request.
